# Systemic Antisense Therapeutics for Dystrophin and Myostatin Exon Splice Modulation Improve Muscle Pathology of Adult *mdx* Mice

**DOI:** 10.1016/j.omtn.2016.11.009

**Published:** 2016-12-10

**Authors:** Ngoc Lu-Nguyen, Alberto Malerba, Linda Popplewell, Fred Schnell, Gunnar Hanson, George Dickson

**Affiliations:** 1 School of Biological Sciences, Royal Holloway-University of London, Egham, Surrey TW20 0EX, UK; 2 Sarepta Therapeutics Inc., 215 First Street, Cambridge, MA 02142, USA

**Keywords:** antisense oligonucleotides, Duchenne muscular dystrophy, dystrophin, exon skipping, myostatin

## Abstract

Antisense-mediated exon skipping is a promising approach for the treatment of Duchenne muscular dystrophy (DMD), a rare life-threatening genetic disease due to dystrophin deficiency. Such an approach can restore the disrupted reading frame of dystrophin pre-mRNA, generating a truncated form of the protein. Alternatively, antisense therapy can be used to induce destructive exon skipping of myostatin pre-mRNA, knocking down myostatin expression to enhance muscle strength and reduce fibrosis. We have reported previously that intramuscular or intraperitoneal antisense administration inducing dual exon skipping of dystrophin and myostatin pre-mRNAs was beneficial in *mdx* mice, a mouse model of DMD, although therapeutic effects were muscle type restricted, possibly due to the delivery routes used. Here, following systemic intravascular antisense treatment, muscle strength and body activity of treated adult *mdx* mice increased to the levels of healthy controls. Importantly, hallmarks of muscular dystrophy were greatly improved in mice receiving the combined exon-skipping therapy, as compared to those receiving dystrophin antisense therapy alone. Our results support the translation of antisense therapy for dystrophin restoration and myostatin inhibition into the clinical setting for DMD.

## Introduction

Duchenne muscular dystrophy (DMD) is the most common fatal muscular disease in children, affecting approximately one in 3,500 male births.^1^ This X-linked recessive disorder is characterized by the absence of dystrophin protein due to mutations in the *DMD* gene.^2^ Dystrophin provides a crucial structural connection among the muscle cytoskeleton, the sarcolemma, and the extracellular matrix to maintain muscle integrity.^3^, ^4^ The absence of dystrophin makes myofibers extremely susceptible to injury during muscle contraction, which leads to progressive muscle deterioration and weakness, respiratory insufficiency, cardiac failure, and premature death.^5^, ^6^

Since the identification of the genetic cause of DMD almost 30 years ago,^2^ many strategies have been developed for symptomatic treatment of the disease, but none has yet proven to be curative. Current therapies are able to address several dystrophinopathy symptoms to improve the quality of life for DMD patients or delay the disease development, but they fail in halting the progression completely.^7^, ^8^, ^9^, ^10^ Gene- and cell-based approaches, on the other hand, provide promise for a cure, as they have shown abilities to correct the faulty *DMD* gene,^11^, ^12^ to add a modified form of the *DMD* gene,^13^, ^14^, ^15^, ^16^ or to generate myofibers from engrafted mesoangioblasts.^17^ Among these, antisense therapy has been considered as one of the most promising approaches,^18^, ^19^ and so far it is the only genetic therapy to be conditionally approved by the FDA for DMD treatment (i.e., EXONDYS 51, Eteplirsen, Sarepta Therapeutics). The approach uses small antisense oligonucleotides designed to silence enhancer motifs on out-of-frame exons in the *DMD* pre-mRNA to restore the *DMD* reading frame and recover production of dystrophin protein, in a shortened but functional form.^20^ Dystrophin restoration solely has slowed down the disease progression in many animal models of DMD.^21^, ^22^, ^23^ However, such an approach suffers the limitation of DMD being often diagnosed when skeletal muscles are severely wasted and only a minor portion of muscle tissue remains. Furthermore, multiple problems that developed in advanced stages of the disease (i.e., muscle infiltration with fat and connective tissue, respiratory and cardiac dysfunction, and reduced muscle function as a consequence of substantial muscle fiber loss^6^, ^24^, ^25^, ^26^, ^27^, ^28^) are very challenging for this treatment. Hence, several adjunctive therapies have been investigated recently, in particular for enhancing muscle strength and reducing fibrosis. One of the most promising strategies is targeting the myostatin signaling.

Myostatin is a negative regulator of skeletal muscle growth and differentiation,^29^ an enhancer of muscle fibroblast proliferation,^30^ and an indirect modulator of adipogenesis.^31^ Myostatin downregulation has been reported to increase muscle mass and muscle strength in an *mdx* mouse model of DMD through the use of myostatin-blocking agents like monoclonal antibodies,^32^, ^33^ recombinant myostatin propeptides,^34^, ^35^ myostatin antagonists,^36^, ^37^ or soluble myostatin receptors.^38^ We and others have demonstrated that it is possible to employ antisense therapy inducing destructive exon skipping of myostatin pre-mRNA for inhibiting myostatin expression. This strategy provided effective myostatin skipping in human and murine dystrophic cell cultures^39^ and increased muscle mass in wild-type mice.^40^ Combinatorial therapy with an antisense approach restoring dystrophin in *mdx* mice, through intramuscular^41^ or intraperitoneal injection,^22^ enhanced the therapeutic benefits offered by dystrophin restoration alone.

Here we performed intravenous systemic delivery of phosphorodiamidate morpholino oligomers conjugated with B peptide (BPMOs), an arginine-rich cell-penetrating peptide, for open reading frame rescue of dystrophin and destructive exon skipping of myostatin. Following 10 consecutive weeks of treatment, treated *mdx* mice displayed an increase in muscle strength comparable to levels of wild-type mice, associated with amelioration of dystrophic pathology. Importantly, our data demonstrate enhanced therapeutic benefits when body-wide dystrophin restoration is combined with myostatin inhibition compared to the single dystrophin therapy.

## Results

### Combined Antisense Therapy Counteracts Pathological Muscle Pseudohypertrophy in Treated mdx Mice

**Figure 1:**
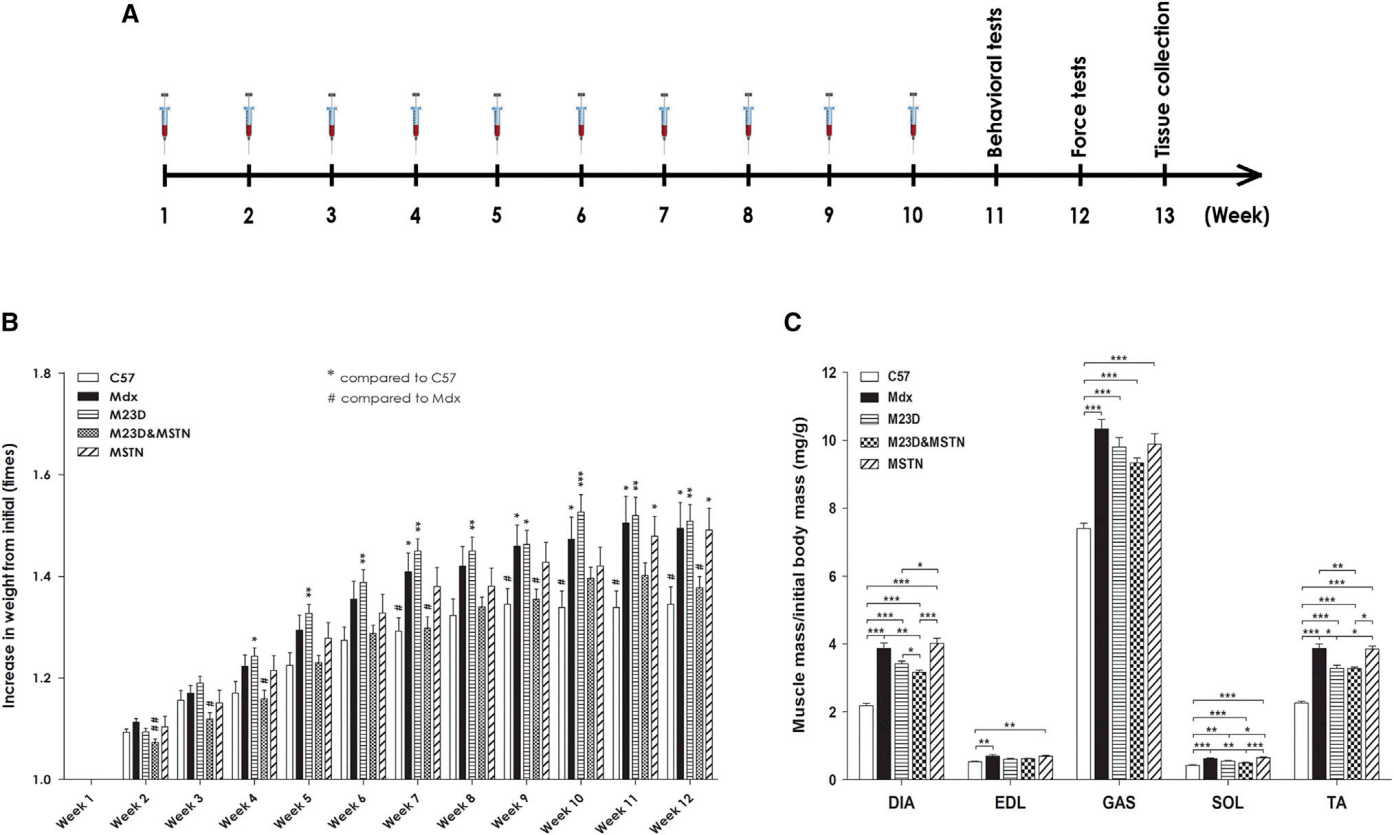
Combined BPMO Treatment Has Beneficial Effects on Body and Muscle Weight of Treated mdx Mice (A) Experimental design describing weekly administration of either BPMOs or saline via intravenous tail vein injection. Animal behavior and forelimb strength were assessed during weeks 11 and 12, followed by tissue collection on the week after. (B) Body weight recorded every week was normalized to the initial weight. (C) Muscle mass of DIA, EDL, GAS, SOL, and TA was evaluated and normalized to the initial body weight. Data in (B) and (C) are expressed as means ± SEM; error bars represent the SEM; n = 10/group. Statistical comparison in each muscle type was by one-way ANOVA followed by Bonferroni’s post hoc test. Significance levels were set at *p < 0.05, **p < 0.01, and ***p < 0.001. The control group in (B) was either C57 or untreated mdx.

Forty 6-week-old *mdx* male mice were initially randomized into four groups matched for average body weight. Animals were injected intravenously with phosphorodiamidate morpholino oligomer (PMO) conjugated to a cell-penetrating peptide (see Materials and Methods). Dystrophin-restoring BPMO targets exon 23 in the mouse dystrophin gene and the MSTN-inhibitory BPMO targets exon 2 in the myostatin gene (BPMO-M23D and BPMO-MSTN, respectively). Mice received either 10 mg/kg BPMO-M23D (n = 10), 10 mg/kg BPMO-MSTN (n = 10), a cocktail of 10 mg/kg BPMO-M23D and 10 mg/kg BPMO-MSTN referred to as BPMO-M23D&MSTN (n = 10), or volume-matched sterile saline (n = 10). An age-matched C57 male group (n = 10) receiving an equivalent volume of sterile saline acted as non-*mdx* strain control. BPMOs or saline was administered weekly through tail vein intravenous injection for 10 consecutive weeks (Figure 1A). Body weight was recorded every week and normalized to the initial body weight (Figure 1B). Muscles of *mdx* mice present pathological muscle pseudohypertrophy due to fiber branching and chronic cycles of muscle degeneration/regeneration associated with an increase of small centrally nucleated fibers.^24^, ^42^ As a consequence, muscle and body weights of *mdx* mice are heavier than the weights of C57 controls. Following 10-week BPMO treatment, however, *mdx* mice receiving BPMO-M23D&MSTN displayed a significant reduction in weight compared to untreated or BPMO-M23D-treated mice. Such weight loss was immediate, maintained until the end of the experiment, and of a magnitude that restored *mdx* body weight to wild-type levels. No change in body weight was detected in BPMO-M23D- or BPMO-MSTN-treated animals compared to saline-injected *mdx* mice.

Two weeks after the last injection, diaphragm (DIA), extensor digitorum longus (EDL), gastrocnemius (GAS), soleus (SOL), and tibialis anterior (TA) muscles were harvested. Muscle weight was normalized to the initial body weight (Figure 1C). In all muscle groups analyzed, muscle mass of BPMO-MSTN- or saline-injected *mdx* was heavier than the mass of C57 muscles. On the contrary, DIA, SOL, and TA muscles of mice treated with BPMO-M23D&MSTN were significantly lighter than muscles of saline-injected *mdx* mice (p = 0.006, 0.006, and 0.04, respectively). Furthermore, we observed a significant reduction in DIA mass (p = 0.03) and a downward trend in GAS mass (p = 0.15) of the group treated with BPMO-M23D&MSTN, compared to those harvested from BPMO-M23D-treated mice.

These data suggest that the combined BPMO-M23D&MSTN antisense therapy may have a beneficial effect on counteracting muscle pseudohypertrophy, typical of *mdx* mice, particularly during the early stage of the disease. Such an effect importantly lasted for at least until the end of the experiment and appeared predominantly in DIA muscle. Although we only analyzed some representative muscles of the body, we expected a similar outcome in other muscle types contributing to the significant amelioration in the *mdx* pseudohypertrophy, compared to the effect seen with the single BPMO-M23D treatment.

### Efficient Exon Skipping of Dystrophin and Myostatin Pre-mRNAs following BPMO-M23D and BPMO-MSTN Administration, Respectively

**Figure 2:**
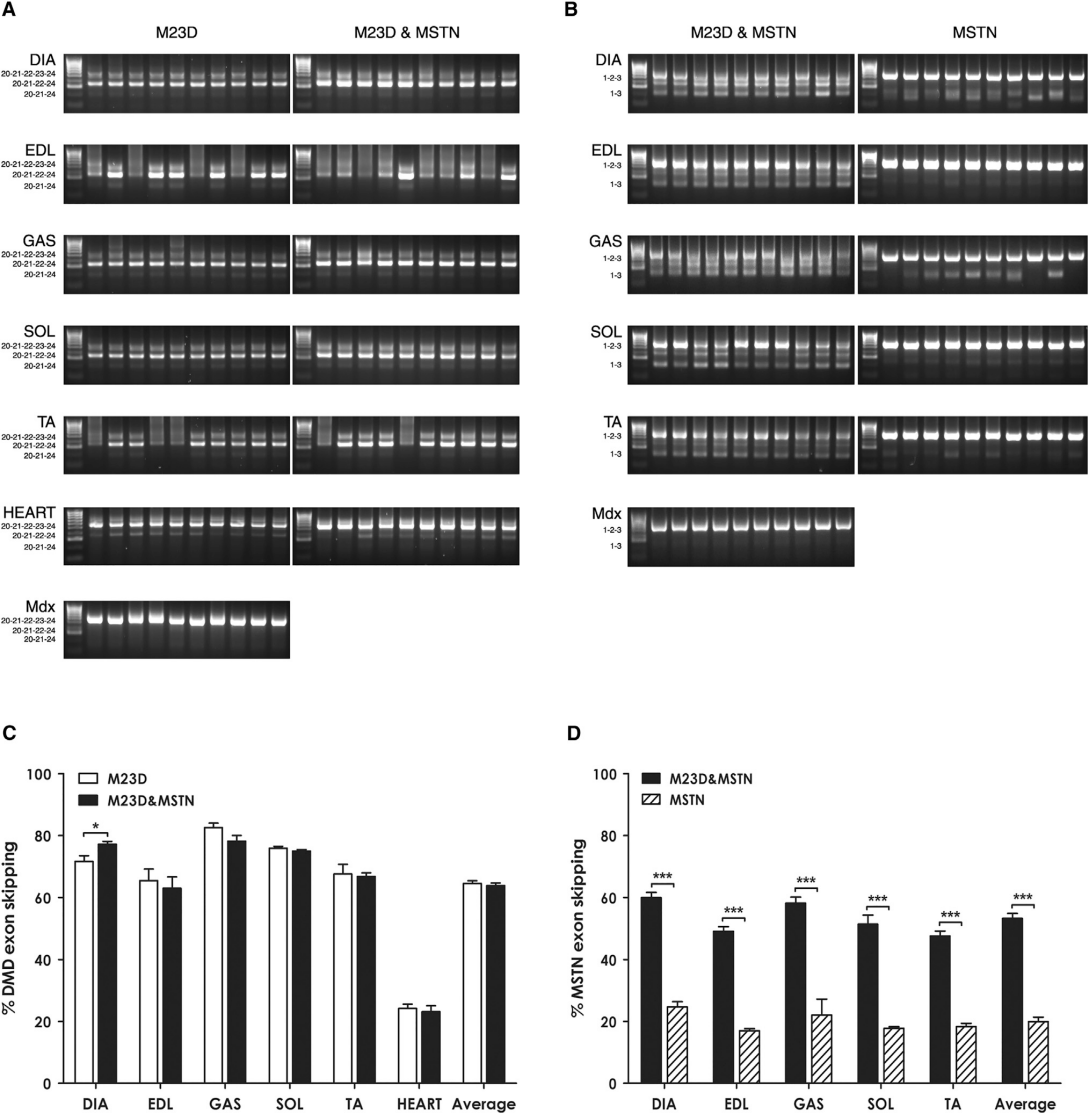
BPMO Delivery Induces Efficient Exon Skipping of Dystrophin and Myostatin Pre-mRNAs (A and B) Gel electrophoresis results show dystrophin and myostatin exon skipping, respectively, in BPMO-treated muscles. Total RNA from DIA, EDL, GAS, SOL, TA, and heart muscles was isolated for semi-nested dystrophin or nested myostatin RT-PCRs. PCR products were loaded in 2% agarose gel. Each lane displays the result from an individual muscle. HyperLadder IV was used as a molecular size standard. Exons included in each band of PCR products are shown to the left of the gels. (C and D) Levels of exon skipping in individual muscles and averaged skipping of all muscles are displayed. The skipping efficiency for (C) dystrophin or (D) myostatin was evaluated through densitometric analysis of RT-PCR products, as a percentage of the density of skipped products compared to the density of both skipped and unskipped products. Data are expressed as means ± SEM; error bars represent the SEM; n = 10/group. Statistical analysis was two-tailed Student’s t test (*p < 0.05 and ***p < 0.001).

Two weeks after the last injection, DIA, EDL, GAS, SOL, TA, and heart muscles were collected from treated mice and processed for RNA extraction and RT-PCR evaluation of dystrophin exon 23 and myostatin exon 2 skipping. RT-PCR demonstrated efficient skipping of both exons in all tissues analyzed (Figures 2A and 2B). Further densitometric analysis of gel electrophoresis results showed that the percentage of the skipped dystrophin pre-mRNA in different muscles ranged between 65.5% ± 11.7% and 82.6% ± 4.6% in the BPMO-M23D treatment and between 63.0% ± 11.6% and 78.2% ± 5.9% in the BPMO-M23D&MSTN treatment. Notably, skipping efficacy in cardiac muscles from single and dual treatments was 24.2% ± 4.3% and 23.2% ± 5.9%, respectively (Figure 2C). In the same muscles, the efficiency of myostatin pre-mRNA skipping was lowest at 17.0% ± 1.5% or 47.6% ± 5.1% and highest at 24.7% ± 5.3% or 60.0% ± 5.4% in the single or dual treatment, respectively (Figure 2D). The combined treatment induced significantly (p = 0.04) higher dystrophin exon skipping in DIA muscle (7% increase) and MSTN exon skipping in all the muscles analyzed (250% increase) compared to the single BPMO-M23D or BPMO-MSTN treatment, respectively (Figures 2C and 2D).

### BPMO-M23D Provides Substantial Body-wide Dystrophin Restoration that Is Markedly Enhanced by BPMO-MSTN Co-administration

**Figure 3:**
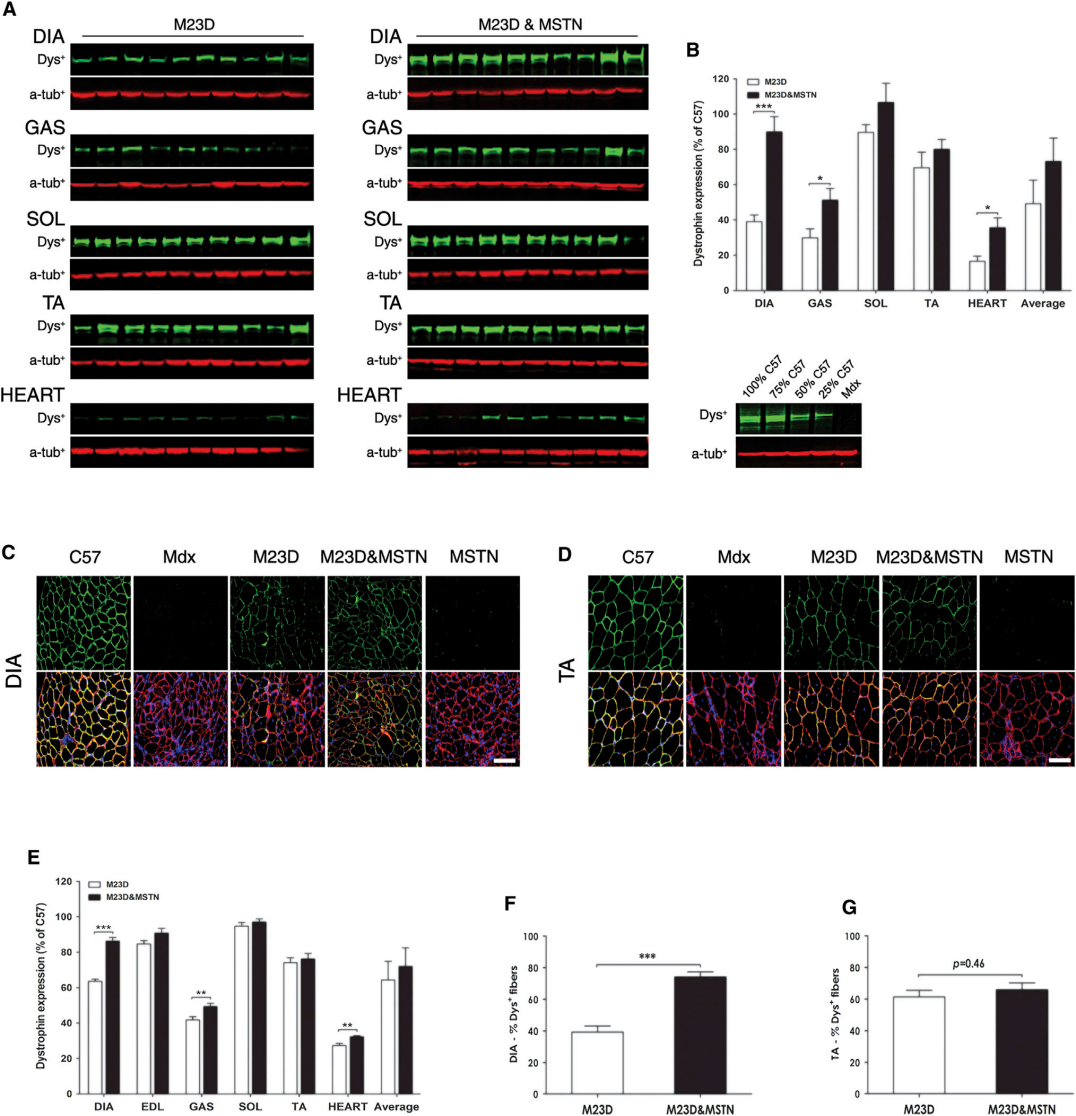
BPMO-M23D and BPMO-M23D&MSTN Administration Induce Substantial Body-wide Dystrophin Restoration (A) Western blot analysis showing dystrophin expression (dys^+^) in DIA, GAS, SOL, TA, and heart muscles of BPMO-M23D- and BPMO-M23D&MSTN-treated mdx mice. Each lane represents a sample from an individual mouse. Alpha-tubulin (α-tub^+^) was used as an internal loading control for western blot. (B) Quantification of dystrophin expression by densitometric analysis of western blot. Following western blot evaluation, the intensity of dys^+^ patterns was scored and normalized to the intensity of corresponding α-tub^+^ patterns, and subsequently it was quantified based on a standard curve of C57 dystrophin. The results were expressed as the percentage of muscle type-matched C57 value (considered as 100%). Data are shown for individual muscle types or as an average of all types. (C and D) Immunostaining detecting dystrophin and laminin expression in treated muscles. Representative images of (C) DIA and (D) TA muscle sections for each group of mice are shown, respectively. Dystrophin-positive fibers were stained in green while laminin-positive fibers were stained in red. Nuclei were stained in blue with DAPI. Scale bars, 100 μm. (E) Quantification of dystrophin intensity levels in DIA, EDL, GAS, SOL, TA, and heart muscles. Following immunostaining for dystrophin, the mean dystrophin intensity was scored by ZEN software and normalized to the mean intensity of laminin detected on the same section. Results were expressed as the percentage of C57 value, considered as 100%. (F and G) Quantification of dystrophin-positive fibers was focused on (F) DIA and (G) TA muscles. The number of dystrophin- and laminin-positive fibers from five random fields of mid-belly muscle sections was counted. Only fibers showing continuous staining of dystrophin along the entire sarcolemma were considered as dystrophin positive and evaluated as the percentage of the number of total fibers (laminin positive) within the same image field. Results were expressed as the percentage of muscle type-matched C57 value, obtained in the same way and considered as 100%. Data in (B) and (E)–(G) are shown as means ± SEM; error bars represent the SEM; n = 10/group. Statistical comparison was two-tailed Student’s t test (*p < 0.05, **p < 0.01, and ***p < 0.001).

DIA, GAS, SOL, TA, and heart tissues were processed for protein extraction, and immunoblot for dystrophin was performed (Figure 3A). Dystrophin expression was quantified by densitometric analysis of protein bands, normalized to the level of endogenous α-tubulin, and it was given as the percentage of dystrophin level detected in C57 muscles (plotted against the standard curve of wild-type dystrophin, see Materials and Methods). Muscles treated with BPMO-M23D and BPMO-M23D&MSTN expressed an average of 49.2% ± 13.4% and 73.2% ± 13.2% dystrophin, respectively (Figure 3B). The combined antisense treatment significantly increased the level of dystrophin protein restored in GAS (p = 0.020) and heart (p = 0.016) muscles. Particularly, the dystrophin level expressed in DIA muscle was 2-fold higher than the level quantified in mice receiving the BPMO-M23D treatment, reflecting the enhanced dystrophin skipping observed by RT-PCR. Dystrophin expression in transverse DIA, EDL, GAS, SOL, TA, and heart sections was additionally measured following immunofluorescence (Figures 3C, 3D, and S1). As expected, very strong dystrophin expression was observed in C57 samples, whereas only few revertant dystrophin-positive fibers were detected in muscles of saline- or BPMO-MSTN-treated *mdx* mice. Substantial expression of dystrophin was observed in muscles of both BPMO-M23D- and BPMO-M23D&MSTN-treated mice at 64.3% ± 25.7% and 72.0% ± 25.7%, respectively (Figure 3E). Consistent with the dystrophin expression observed by western blot analysis, the level of epifluorescence detected by dystrophin immunostaining was significantly higher in DIA, GAS, and heart muscles (p = 0.0001, 0.006, and 0.001, respectively) of mice treated with the combined BPMOs, compared to single BPMO-M23D treatment.

Since DMD patients mostly die due to respiratory failure, rescue of DIA function is crucial in DMD treatment. Thus, we focused on evaluating the therapeutic efficacy in DIA muscle and extended to TA muscle, which is commonly examined in DMD research. The numbers of dystrophin-positive fibers in DIA and TA muscle sections were counted, normalized to the total fiber numbers of the same sections, and shown as percentages of C57 controls. An average of 7,800 DIA myofibers and 3,500 TA myofibers from each treated group were assessed. A higher number of dystrophin-positive fibers was seen in the DIA of mice treated with the combined BPMOs compared to the muscles of BPMO-M23D-injected mice (p = 0.0001) (Figure 3F), which correlated well with dystrophin expression detected by western blot and immunostaining. In TA muscle, there was a comparable level (p = 0.46) of dystrophin expression between the single and dual treatments, with over 60% detectable dystrophin-expressing fibers (Figure 3G). Taken together, these results demonstrate that BPMO-M23D delivery efficiently rescued body-wide dystrophin expression. Importantly, the efficacy was enhanced, particularly in DIA muscle, by co-administration of BPMOs downregulating myostatin levels.

### Antisense Therapy Rescuing Dystrophin Expression Ameliorates Dystrophic Hallmarks in mdx Mice, with or without Myostatin Inhibition

**Figure 4:**
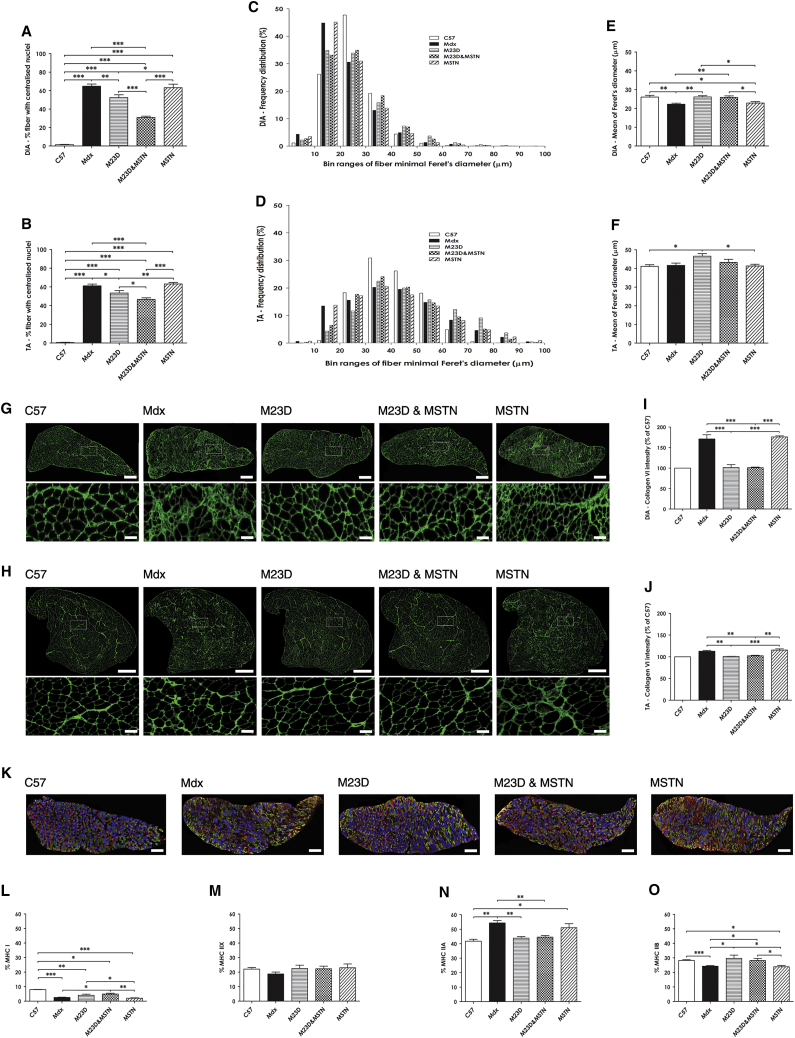
BPMO-Mediated Therapy Robustly Improves Hallmarks of Dystrophic Muscles (A and B) Quantification of centrally nucleated fibers in (A) DIA and (B) TA muscles, respectively. Results are expressed as the percentage of the total fibers. (C and D) Frequency distribution of the minimal Feret’s diameter of (C) DIA and (D) TA myofibers. Muscle sections were immunostained for laminin. The minimal Feret’s diameter was semi-automatically measured by ZEN imaging analysis software. Incomplete fibers were excluded from the analysis. The frequency distribution of the Feret’s diameter was analyzed by Prism5. Data are shown as the percentage of the total fiber number. (E and F) Mean of the Feret’s diameter is displayed for (E) DIA and (F) TA fibers, respectively. (G and H) Evaluation of muscle fibrosis in (G) DIA and (H) TA cross sections. Immunostaining for collagen VI was performed. Representative mosaic images showing the entire sections and images at higher magnification are shown. Scale bars, 100 μm (enlarged images of both G and H), 500 μm (G), and 1,000 μm (H). (I and J) Quantification of muscle fibrosis. Following immunostaining for collagen VI, the mean intensity of collagen VI was measured by ZEN software and expressed as the percentage of C57 values (considered as 100%). (K) Immunostaining of DIA sections using antibodies detecting four MHC fiber types. Representative mosaic images of all treatment groups are shown. MHC I fibers were stained in red, MHC IIA fibers were stained in green, MHC IIB fibers were stained in blue, and MHC IIX fibers were unstained. Immunostaining for laminin was used for identifying the sarcolemma of the myofibers. Scale bars, 500 μm. (L–O) Quantification of MHC fibers in DIA transverse sections. Following immunostaining, mosaic images of the whole-muscle sections were generated using ZEN software. The number of MHC-positive fibers was counted separately using ImageJ software, and it was expressed as the percentage of the total number of all fiber types within each muscle section. Data in (A), (B), (E), (F), (I), (J), and (L)–(O) are shown as means ± SEM; error bars represent the SEM; n = 10/group. Statistical comparison was by one-way ANOVA followed by Bonferroni’s post hoc test (*p < 0.05, **p < 0.01, and ***p < 0.001).

Since dystrophic muscle fibers are susceptible to necrosis and undergo repeated degeneration-regeneration cycles, an important hallmark of the dystrophic phenotype is increases in centrally nucleated muscle fibers (CNFs). Hence, we calculated the amount of CNFs in DIA and TA muscle sections as a percentage of the total fiber number (Figures 4A and 4B, respectively). As expected, only a few CNFs were detected in healthy C57 muscles, while the percentage in muscles of saline- or BPMO-MSTN-treated groups was over 60%. Treatment with BPMO-M23D or BPMO-M23D&MSTN lowered the amount of CNFs to 52.5% ± 12.7% and 30.9% ± 6.5% in DIA muscle and to 53.1% ± 12.1% and 46.7% ± 8.0% in TA muscle, respectively. As shown, the amount of CNFs was significantly lower in the combined treatment compared to the single BPMO-M23D administration (p = 0.0001).

CNFs are intrinsically smaller than mature muscle fibers. Therefore, a therapeutic strategy would be expected to protect the fibers from degeneration and to increase the average myofiber size of treated muscles. Immunostaining for laminin was used to delineate the sarcolemma and enable morphometric measurement of the relative size of muscle fibers.^4^ The frequency of distribution of the minimal Feret’s diameter demonstrated a shift in the distribution of DIA and TA myofibers of BPMO-M23D- and, in particular, of BPMO-M23D&MSTN-treated mice toward the values of C57 controls (Figures 4C and 4D, respectively). These results were confirmed by the analysis of the mean Feret’s diameter of the muscles (Figures 4E and 4F). Muscles treated with BPMO-M23D or with BPMO-M23D&MSTN displayed an increase in the mean of the Feret’s diameter that is directly correlated to an increase in muscle fiber cross-sectional area. Treatment with BPMO-MSTN alone had no effect on the muscle fiber size, suggesting that restoration of functional truncated dystrophin to suppress muscle degeneration/regeneration processes is essential to obtain maximum beneficial effects of myostatin downregulation.

The formation of excess fibrous connective tissue is one of the most important hallmarks of dystrophic muscles. By immunostaining for collagen VI, a component of endomysial connective tissue, we assessed the level of fibrosis in DIA and TA muscles (Figures 4G and 4H, respectively). Collagen VI epifluorescence intensity was quantified and expressed as a percentage of the level detected in muscles of C57 mice (Figures 4I and 4J). The results indicated substantial muscle fibrosis in DIA muscles and a less severe but clearly detectable fibrosis in TA muscles of saline- or BPMO-MSTN-injected *mdx* mice. In contrast, administration of BPMO-M23D alone or in combination with BPMO-MSTN prevented the formation of fibrosis in both muscle types, lowering the amount of collagen VI detected to the level of wild-type muscles.

Switching between myofiber types is a further indicator of ongoing dystrophic pathophysiology. Since muscle fibers express various muscle protein isoforms, i.e., myosin heavy chain (MHC), to identify the fiber-type composition of DIA muscles, we immunostained the muscles for four MHC isotypes (Figure 4K), and subsequently we quantified the number of four major myofiber types (expressed as percentages of the total number of fibers). All *mdx* muscles displayed a reduction in type I fibers (Figure 4L) but no significant change in type IIX fibers (p = 0.61) (Figure 4M), as compared to muscles of C57 mice. However, in comparison to saline-injected *mdx* muscles, treatment with BPMO-M23D&MSTN significantly increased the level of type I fibers (p = 0.02), while BPMO-M23D treatment provided a trend in this fiber type (p = 0.08). The percentage of type IIA (Figure 4N) or type IIB (Figure 4O) fibers in BPMO-M23D- or BPMO-M23D&MSTN-treated muscles was normalized to the wild-type level, while BPMO-MSTN treatment was less efficient at doing so.

Overall, the data demonstrate an improvement in several histopathological hallmarks of dystrophic muscles following BPMO-mediated dystrophin recovery. Importantly, the results further indicate that combining myostatin inhibition and dystrophin restoration enhanced therapeutic effects of the antisense therapy, mainly in the respiratory DIA muscle.

### Combining Myostatin Inhibition and Dystrophin Restoration Normalizes Muscle Strength to Wild-Type Level and Improves Animal Behavior

**Figure 5:**
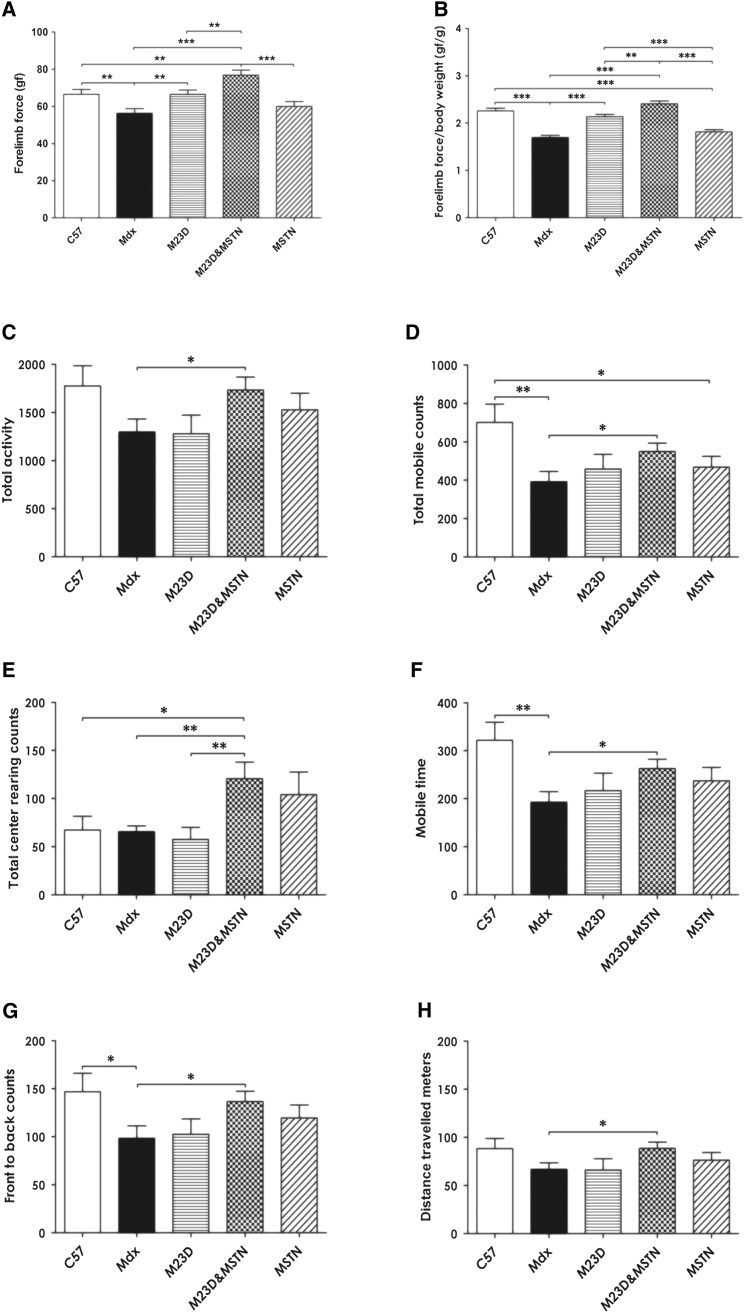
Effects of Antisense Therapy on Muscle Strength and Animal Behavior (A) Evaluation of forelimb muscle force by grip strength test. Assessment was performed 1 week after the last injection of BPMOs or saline. (B) Forelimb strength was normalized to the final body weight and expressed as gram force per gram of body weight. (C–H) Mouse open-field behavioral activity. Assessment was performed using locomotor activity monitors. Representative parameters of the animal behavior are shown as arbitrary units. Data are shown as means ± SEM; error bars represent the SEM; n = 10/group. Statistical significance was by one-way ANOVA followed by Bonferroni’s post hoc test (*p < 0.05, **p < 0.01, and ***p < 0.001).

Prior to harvesting muscles, mice underwent functional tests to assess the effect of BPMO treatments on muscle strength. The force generated by the forelimbs was measured by grip strength test (Figure 5A) and normalized to the final body weight (Figure 5B). BPMO-MSTN- or saline-injected *mdx* mice were significantly weaker than C57 mice (20% and 25% of the level measured in wild-type mice, respectively). On the contrary, the administration of BPMO-M23D or BPMO-M23D&MSTN rescued the forelimb strength of treated *mdx* to the wild-type level. Notably, forelimbs of BPMO-M23D&MSTN-treated *mdx* mice were statistically significantly stronger than the forelimbs of BPMO-M23D-treated animals (p = 0.005).

Animal locomotor behavior was further assessed using open-field behavioral activity monitoring cages. A total of 22 parameters describing the animals’ activity were analyzed (Table S1). In six of 22 parameters, saline-injected *mdx* mice showed statistically significant behavior differences compared to wild-type mice. All six parameters were reverted to wild-type levels in animals receiving single BPMO-M23D or BPMO-M23D&MSTN cocktail treatment. However, in comparison between treated and non-treated *mdx* mice, only those receiving BPMO-M23D&MSTN treatment displayed significant changes (13 of 22 parameters) versus saline-treated animals, all toward wild-type-like behavior. This demonstrates the benefit of co-administration of BPMOs to downregulate myostatin and rescue dystrophin transcripts. Notably, mice receiving the combined BPMO-M23D&MSTN exhibited an increase in rearing, superior to the level of wild-type mice (Figures 5C–5H; Table S1), which suggests a possible increase in the hindlimb strength of these animals. These data demonstrate that the combined BPMO-M23D&MSTN treatment improves muscle strength and the general body activity of the BPMO-M23D approach.

## Discussion

Our study on systemic antisense therapy for dystrophin restoration and myostatin inhibition demonstrated efficient rescue of body-wide dystrophin expression, notably in crucial muscles like the diaphragm and the heart. As a consequence, the dual treatment ameliorated the pathology of DMD and increased body activity and muscle strength of treated *mdx* mice, significantly improving the effect of the single dystrophin approach. Strikingly, while we expected to observe muscle hypertrophy following myostatin knockdown, the body mass and muscle mass of treated animals decreased toward the wild-type values. Moreover, the therapy based on only myostatin inhibition offered no improvement in any of the parameters examined. A likely explanation is that myostatin knockdown provided by systemic delivery of BPMOs might be effective to show some beneficial effect on the pathology but insufficient to provide muscle hypertrophy in a dystrophic background. Muscle degeneration due to a lack of dystrophin is compensated for by innate muscle regeneration in the early weeks of the *mdx* lifetime.^24^ Such a phenomenon is associated with myofiber branching^43^ that results in the typical dystrophic pseudohypertrophy. Branched myofibers are weaker and more susceptible to damage than unbranched fibers, and once formed they do not fuse with parent myofibers.^44^, ^45^ Hence, a possible explanation for the lack of muscle hypertrophy is that myostatin knockdown is only effective in wild-type-like myofibers and may be insufficient in rescuing branched myofibers. This is further supported by previous studies that showed that destructive myostatin exon skipping successfully induced muscle hypertrophy in treated wild-type muscles^40^ and was limited to smaller female *mdx* muscles, where the effect of the treatment could be seen more obviously.^41^

Numerous studies, however, have reported an increase in muscle mass and muscle strength of *mdx* mice following myostatin downregulation in the absence of dystrophin restoration. The authors used either recombinant myostatin antibodies,^32^, ^33^ myostatin propeptides,^34^, ^35^ myostatin antagonists,^36^, ^37^ or soluble myostatin receptors.^38^ By targeting myostatin at the protein level, the effects from these strategies were immediate and generally more efficient than using antisense therapies to disrupt myostatin pre-mRNA.^46^ Indeed, a reduction in myostatin synthesis in skeletal muscles due to destructive exon skipping can be compensated for by the reactivation of circulating myostatin from its latent form in the bloodstream,^47^ whereas therapies blocking the myostatin protein also act on circulating myostatin and do not present this issue. Why we did not observe muscle mass increase remains a matter of further investigation. This also could be due to the dose regimen used, the administration frequency, or the length of the treatment.

Although it appears that systems acting on the protein are more efficient to knock down myostatin than systemic exon skipping by BPMOs, the main drawback of myostatin protein blockade is the related strong off-target side effects. Clinical trials for DMD using soluble myostatin receptors have reported adverse events and eventually have been terminated due to potential safety concerns of epistaxis and telangiectasia (https://clinicaltrials.gov; NCT01239758 and NCT01099761). Furthermore, since myostatin has a significant homology and shares the signaling pathway with many members of the transforming growth factor β (TGF-β) family, protein blockade is clearly not specific to myostatin but also affects other proteins of this family. For instance, growth and differentiation factor 11 (GDF11), an inhibitor of skeletal muscle differentiation that has 90% homology with myostatin in the mature active region,^48^ potentially is inhibited by myostatin antibodies, myostatin propeptides, or myostatin antagonists such that the effects seen on muscle hypertrophy cannot be considered to be a result of myostatin knockdown alone.^49^ Treatment with follistatin, a myostatin antagonist, effectively enhanced the mass of *mdx* muscles.^36^ However, the inhibitory effect also had an influence on multiple tissues other than the skeletal muscle due to additional blockade of activin, another close relative of myostatin.^50^ On the other hand, exon skipping therapy is specific for myostatin because the strategy employs antisense oligonucleotides that are specifically designed based on the sequence of the *Mstn* gene; therefore, this approach can minimize the off-target side effects observed in protein blockade therapies. This is crucial when translating a potentially therapeutic approach for DMD to clinical use, as the safety of such a long-term treatment needs to be prioritized. Additionally, the antisense approach provides many advantages, such as flexibility in dosage; frequency of dosing; transient effect in nature; and, importantly, a possible preferential path by regulatory bodies over other strategies due to the recent FDA conditional approval of antisense treatment for exon 51 (EXONDYS 51).

The diaphragm of young *mdx* mice exhibits highly severe and progressive muscle degeneration and fibrosis similar to limb muscles of DMD boys.^24^, ^51^ A restoration of more than 40% of dystrophin protein level following BPMO-M23D administration was sufficient to prevent the formation of fibrosis in the treated muscles. Therefore, an additional knockdown of myostatin in the combined treatment, with increased dystrophin recovery, obviously could not provide further reduction in the fibrosis level. Despite that myostatin is an enhancer of muscle fibroblast proliferation^30^ and myostatin knockdown was expected to suppress muscle fibrosis, the single BPMO-MSTN approach surprisingly did not decrease diaphragm fibrosis. Consistent with previous observation, this suggests a possible limitation of the myostatin destructive antisense approach in the pre-clinical dystrophic mouse model when used as a single therapy.

Lacking in inducing muscle hypertrophy, exon skipping-mediated myostatin knockdown, however, substantially enhanced other therapeutic benefits of the dystrophin restoration approach. The combined treatment led to a significant decrease in the number of centrally nucleated fibers and in changes in the body and muscle mass reverting toward the wild-type properties. Consistent with our previous findings in newborn *mdx* mice,^22^ systemic myostatin knockdown contributed to the recovery of dystrophin expression, particularly in the diaphragm muscle. Restoring the functionality of diaphragm muscle is crucial for DMD as most patients die from respiratory failure.^6^ With support from dystrophin recovery in other skeletal muscles, the dual antisense therapy moreover provided a remarkable improvement on forelimb muscle strength and body activity. Although the mechanism of muscle hypertrophy following myostatin inhibition is unclear, several studies have suggested that myostatin knockdown causes hypertrophy mainly by acting on myofibers, increasing the cytoplasmic volume to the DNA ratio possibly by upregulating the Akt/mTOR/p70S6K protein synthesis pathway, rather than stimulating satellite cells.^52^, ^53^ Thereby, the synthesis rate of cytoplasmic proteins, such as dystrophin, is likely to be elevated when a dystrophin-restorative strategy also is used. This consequently enhances the stability of muscle integrity, stops the repeated cycles of muscle necrosis and regeneration, and repositions nuclei of regenerated myofibers to their normal location at the periphery,^54^ more effectively than the therapy restoring dystrophin alone.

Interestingly, a virtuosos circle was established as not only myostatin knockdown increased dystrophin restoration but also vice versa, as muscles receiving the combined BPMO-M23D&MSTN treatment displayed an increase in exon-skipping efficiency of MSTN pre-mRNA compared to those in the single BPMO-treated groups. Clearly, when myofibers benefited from dystrophin recovery that stabilized the muscle integrity and diminished the depletion of the fibers, the efficiency of exon skipping for both dystrophin and myostatin increased. In contrast, when only MSTN BPMOs were administered, there was no protection of muscle fibers, which resulted in higher turnover with a consequent substantial loss of the BPMOs from muscle tissue. Further investigations need to be carried out to understand the mechanisms explaining the increase in protein synthesis following myostatin knockdown. This observation is potentially relevant as other gene addition or gene upregulation strategies (e.g., approaches based on adeno-associated virus (AAV)-mediated delivery of semi-functional micro- or mini-dystrophin^55^ or based on utrophin upregulation^56^) could benefit from such a transient approach using antisense oligonucleotides for myostatin knockdown.

In conclusion, our study provides clear evidence that the dual antisense therapy combining systemic rescue of dystrophin and knockdown of myostatin expression has additional therapeutic benefits over the single dystrophin therapy. The data hence support a translation of this combinatorial antisense approach in a clinical scenario for DMD treatment.

## Materials and Methods

### PMOs and PMO Conjugates

PMO-DMD (5′-GGCCAAACCTCGGCTTACCTGAAAT-3′) and PMO-MSTN (5′-CAGCCCATCTTCTCCTGGTCCTGGGAAGGT-3′) were synthesized and conjugated to an arginine-rich cell-penetrating peptide (so-called B peptide: RXRRBRRXRRBRXB) at the 3′ end of the PMO by Sarepta Therapeutics. PMO-DMD sequence and a 28-mer version of the PMO-MSTN sequence previously have been shown to be biologically active in inducing skipping of dystrophin exon 23 and myostatin exon 2, respectively.^22^, ^41^ BPMOs were re-suspended in sterile double-distilled (dd) H_2_O and diluted in sterile 0.9% saline (Sigma) at a desired concentration prior to injection.

### Animals and Experimental Design

Animal procedures were performed in accordance with the UK Animals (Scientific Procedures) Act, 1986. *Mdx* (C57BL/10ScSn-Dmdmdx) and C57BL/10 mice were bred in our animal facility and were maintained in a standard 12-hr light/dark cycle with free access to food and water. Mice were weaned at postnatal weeks 4–5 and two to six individuals were housed per cage. Since only males were used, mice within each experimental group (n = 10 per group) were obtained randomly from two to three age-matched litters. Four 6-week-old *mdx* groups were injected with 10 mg/kg BPMO-DMD, 10 mg/kg BPMO-MSTN, a combination of 10 mg/kg BPMO-DMD and 10 mg/kg BPMO-MSTN, or volume-matched sterile saline. An age-matched C57 group receiving an equivalent volume of sterile saline was included as a healthy control. BPMOs or saline was administered weekly through tail vein intravenous injection for 10 consecutive weeks. One week after the last injection, animal locomotor behavior was assessed followed by muscle force evaluation by grip strength tests. Tissue collection was performed on the following week.

### Open-Field Behavioral Assessment

Open-field behavioral activity was evaluated using locomotor activity monitors. Each mouse was acclimatized to the test chamber during an undisturbed 1-hr period per day, for 4 consecutive days. Activity and behavioral assessments were carried out 1 week post-acclimation tests. Mice were acclimatized for 30 min prior to data acquisition collected by Amon Lite software (version 1.4) every 10 min in a 1-hr session. The data acquisition procedure was repeated four times daily. Data obtained from each mouse were averaged and expressed as arbitrary units. During the acquisition, particular care was taken to minimize noise and movement in the test room. Both locomotor activity monitors and Amon Lite software were purchased from Linton Instrumentation.

### Measurement of Forelimb Strength

The forelimb strength was assessed using a commercial grip strength monitor (Linton Instrumentation). Measurements were performed five times per mouse over a 3-day period. Mice were held 2 cm from the base of the tail, allowed to grasp a metal mesh attached to a force transducer with their forepaws. The force produced during a gentle pull, until the mice released their grip, was recorded, with 30 s elapsing between each of five sequential tests per mouse per day. Data were collected manually by reading the values on the monitor display, and they were expressed as gram force (raw data) or as gram force per gram of the final body weight.

### Tissue Collection

From each mouse, the DIA, the EDL, the GAS, the SOL, the TA, and the heart tissues were collected. Tissues from one side of the body were frozen immediately in liquid nitrogen for RNA and protein extraction, while tissues from the other side were embedded in optimal cutting temperature medium (VWR) and subsequently frozen in liquid nitrogen-cooled isopentane (Sigma) for cryosectioning. All samples were kept at −80°C until use.

### RNA Extraction and RT-PCR Quantifying Exon-Skipping Efficiency

RNeasy Fibrous Tissue kit (QIAGEN) was used in RNA extraction. Tissue was homogenized in the lysis buffer provided with the kit at 25 Hz for 2 × 2 min, using a TissueLyser II (QIAGEN). The total RNA was then extracted following the manufacturer’s instructions. RNA quantification was performed on an ND-1000 NanoDrop spectrophotometer (Thermo Scientific). Extracted RNA (500 ng) was reverse transcribed using sequence-specific primers by GoScript Reverse Transcription System (Promega). The cDNA products (4 μl) were used as templates in subsequent semi-nested (dystrophin) or nested (myostatin) PCRs, amplified by GoTaq Polymerase (Promega). The final PCR products were loaded onto 2% agarose gels. HyperLadder IV (Bioline) was used as a size standard. Densitometric analysis of gel electrophoresis results was performed using GeneTools Image Analysis software 4.02 (Syngene). The efficiency of dystrophin or myostatin exon skipping was evaluated as the percentage of the density of skipped products against the total density of unskipped and skipped products. Details of RT-PCR programs and primer sequences (MWG) are available upon request.

### Protein Extraction and Western Blot Quantifying Dystrophin Expression

Tissue was homogenized in lysis buffer (0.15 M NaCl, 0.05 M HEPES, 1% NP-40, 0.5% sodium deoxycholate, 0.1% SDS, and 0.01 M EDTA) containing protease inhibitors (Roche) at 25 Hz for 2 × 2 min on a TissueLyser II (QIAGEN). Following a centrifugation at 13,000 rpm, 10 min, and 4°C, the supernatant was transferred to fresh pre-chilled 1.5-mL tubes. The total protein was quantified by *DC* Protein Assay (Bio-Rad) following the manufacturer’s instructions. Protein samples (100 μg) were resolved on 3%–8% Tris Acetate NuPage gels (Life Technologies). HiMark Pre-stained Protein Ladder (Life Technologies) was used as a size standard. The gels were run at 150 V for 1.5 hr, and subsequently they were transferred to HyBond nitrocellulose membranes (GE Healthcare) at 30 V for 2 hr. Membranes were then incubated with blocking buffer (5% skimmed milk, 1× PBS, 0.2% Tween-20) for 1 hr. An overnight incubation at 4°C with either monoclonal mouse anti-dystrophin 6C5 (1:100, Novocastra Laboratories) or rabbit anti-α-tubulin (1:2,500, Abcam) antibody was carried out, followed by incubation with compatible secondary antibodies (1:10,000, LI-COR Biosciences), goat anti-mouse IRDye800 and goat anti-rabbit IRDye680. The blots were visualized on an Odyssey Infrared Imaging System (LI-COR Biosciences). Densitometric analysis of dystrophin- and α-tubulin-positive bands was performed using ImageJ software (NIH). The values of dystrophin intensity were normalized to the values of corresponding α-tubulin intensity and subsequently quantified based on a standard curve of C57 dystrophin. To obtain this standard curve, different amounts of C57-extracted proteins were mixed with *mdx*-extracted proteins, such that in each 100-μg protein mixture there was 100%, 75%, 50%, 25%, or 0% C57 dystrophin. Reagents were purchased from Sigma unless stated otherwise.

### Laminin and Dystrophin Co-immunostaining

Frozen tissue was cryosectioned on an OTF 5000 cryostat (Bright) at 10-μm thickness through the muscle length. Transverse sections were fixed in ice-cold acetone and blocked in 1% BSA, 1% goat serum, 0.1% Triton X-100, and 1× PBS. Sections subsequently were incubated with rat anti-laminin antibody (1:1,000, Sigma) at 4°C overnight. Slides were washed three times in 1× PBS and 0.05% Tween-20 prior to a 1-hr incubation with goat anti-rat Alexa568 (1:1,000, Life Technologies). Dystrophin was stained using Mouse-on-Mouse Basic kits (Vector Laboratories) following the manufacturer’s instructions. Monoclonal mouse anti-dystrophin 6C5 (1:50, Novocastra Laboratories) and goat anti-mouse Alexa488 (1:1,000, Life Technologies) were used. An additional 15-min staining with 1 μg/mL DAPI (Sigma) was performed prior to mounting in Mowiol 4-88 (Sigma).

### Quantifying Dystrophin Expression on Transverse Muscle Sections

Dystrophin immunostaining was visualized under an inverted fluorescence Axio Observer D1 microscope. Images were taken by an AxioCam MR3 combined with ZEN imaging software. Equipment and software were purchased from Carl Zeiss. Overlapping images from each of the mid-belly muscle sections were captured and stitched automatically to create a mosaic image of the section. The mean dystrophin intensity was then scored by ZEN software and normalized to the mean intensity of laminin staining of the same section. For dystrophin-positive fiber counting, five random fields of each muscle section were captured. Counting was performed manually using ImageJ software (NIH). Only fibers showing continuous staining of dystrophin were considered as dystrophin positive and evaluated as a percentage of the number of total fibers within the same image field that were positive with laminin staining. An average of 7,800 DIA and 3,500 TA fibers per treatment were scored. Data of both dystrophin intensity and dystrophin-positive fibers were expressed as the percentage of the values of C57 samples, considered as 100%.

### Immunofluorescent Evaluation of Muscle Fibrosis

Muscle fibrosis was examined following immunostaining for collagen VI. Muscle sections were blocked in 5% milk, 1× PBS, 0.05% Tween-20 for 1 hr, then incubated in order with rabbit anti-collagen VI (1:300, Abcam) and goat anti-rabbit Alexa488 (1:200, Life Technologies) antibodies, 1 hr per incubation. Mosaic images from each of the mid-belly muscle sections were captured and generated by ZEN software (Zeiss) as described above. The mean intensity of collagen VI was measured by the software and shown as the percentage of C57 values obtained in the same way.

### Fiber-Typing Analysis

Frozen sections were fixed in ice-cold acetone for 10 min, then blocked in Mouse-on-Mouse blocking buffer (Vector Laboratories) supplemented with 1% BSA, 1% goat serum, 0.1% Triton X-100, and 1× PBS. Subsequent incubation with primary (for 2 hr) and secondary (for 1 hr) antibodies was performed. Primary antibodies were mouse anti-MHC antibodies (1:10, DSHB), including BA-D5 for MHC I, SC-71 for MHC IIA, and BF-F3 for MHC IIB; rabbit anti-laminin antibody (1:300, Abcam) was additionally used. Secondary antibodies were goat anti-mouse IgG Alexa568 (1:400, Life Technologies), goat anti-mouse IgG Alexa488 (1:400, Life Technologies), goat anti-mouse IgM Alexa405 (1:200, Abcam), and goat anti-rabbit IgG Alexa568 (1:400, Life Technologies), respectively. Following immunostaining, mosaic images of the whole-muscle sections were generated using ZEN software as previously described. The number of myosin-positive fibers was counted separately using ImageJ software (NIH) and expressed as the percentage of the total number of all fiber types within each of the muscle sections; unstained fibers were considered as type IIX.

### Histological Analysis

Laminin immunostaining (as described above) was used for identifying the fiber perimeter. The minimal Feret’s diameter of averaged 7,800 DIA fibers and 3,500 TA fibers (per animal group) were semi-automatically measured by ZEN imaging analysis software (Zeiss); incomplete fibers touching the edge of each image field were excluded from analysis. Automatic analysis of the frequency distribution of the Feret’s diameter was carried out using Prism5 software (GraphPad). The number of fibers having internal nuclei was counted manually using ImageJ software (NIH) and expressed as the percentage of the total fiber number within that section.

### Statistical Analysis

Data were analyzed by GraphPad Prism5 software and shown as the means ± SEM. Error bars represent the SEM; “n” refers to the number of mice per group. Comparisons of statistical significance were assessed by one-way ANOVA followed by Bonferroni’s post hoc test or by two-tailed Student’s t test. The chi-square test was used to evaluate the frequency distribution of fiber diameter, with statistical comparison at df = 10. Significance levels were set at *p < 0.05, **p < 0.01, and ***p < 0.001.

## Author Contributions

N.L.-N., A.M., L.P., and G.D. conceived and designed the study. N.L.-N. and A.M. performed all experiments. BPMO reagents were designed by L.P. and G.D. and synthesized by F.S. and G.H. All authors contributed to result interpretation and data analysis. N.L.-N. and A.M. wrote the manuscript with input from L.P., G.D., and F.S. All authors read and approved the final manuscript.

## Conflicts of Interest

A patent named Oligomers US 9322019 B2 has been filed by Royal Holloway-University of London and a license has been acquired by Sarepta Therapeutics. F.S. and G.H. are employees of Sarepta Therapeutics.

## Supplemental Information

Document S1. Figure S1 and Table S1

Document S2. Article plus Supplemental Information
