# Supplementary material for: Systemic Antisense Therapeutics for Dystrophin and Myostatin Exon Splice Modulation Improve Muscle Pathology of Adult mdx Mice
Source: Mol Ther Nucleic Acids. 2016 Dec 10;6:15–28. doi: 10.1016/j.omtn.2016.11.009 (PMC5363451; doi:10.1016/j.omtn.2016.11.009)
Supplement: Document S1. Figure S1 and Table S1 [file mmc1.pdf]

OMTN, Volume *6*

Supplemental Information

Systemic Antisense Therapeutics for Dystrophin and Myostatin Exon Splice Modulation Improve Muscle Pathology of Adult *mdx* Mice

Ngoc Lu-Nguyen, Alberto Malerba, Linda Popplewell, Fred Schnell, Gunnar Hanson, and George Dickson

Supplementary Materials

Figure S1. Immunostaining confirming BPMO-mediated dystrophin restoration in skeletal and cardiac muscles. Representative images of EDL, GAS, SOL, and HEART muscles for each treatment group are shown. Dystrophin-positive fibers were stained in green, laminin-positive fibers were stained in red, and nuclei were stained in blue with DAPI. Scale bars: 100 μm.

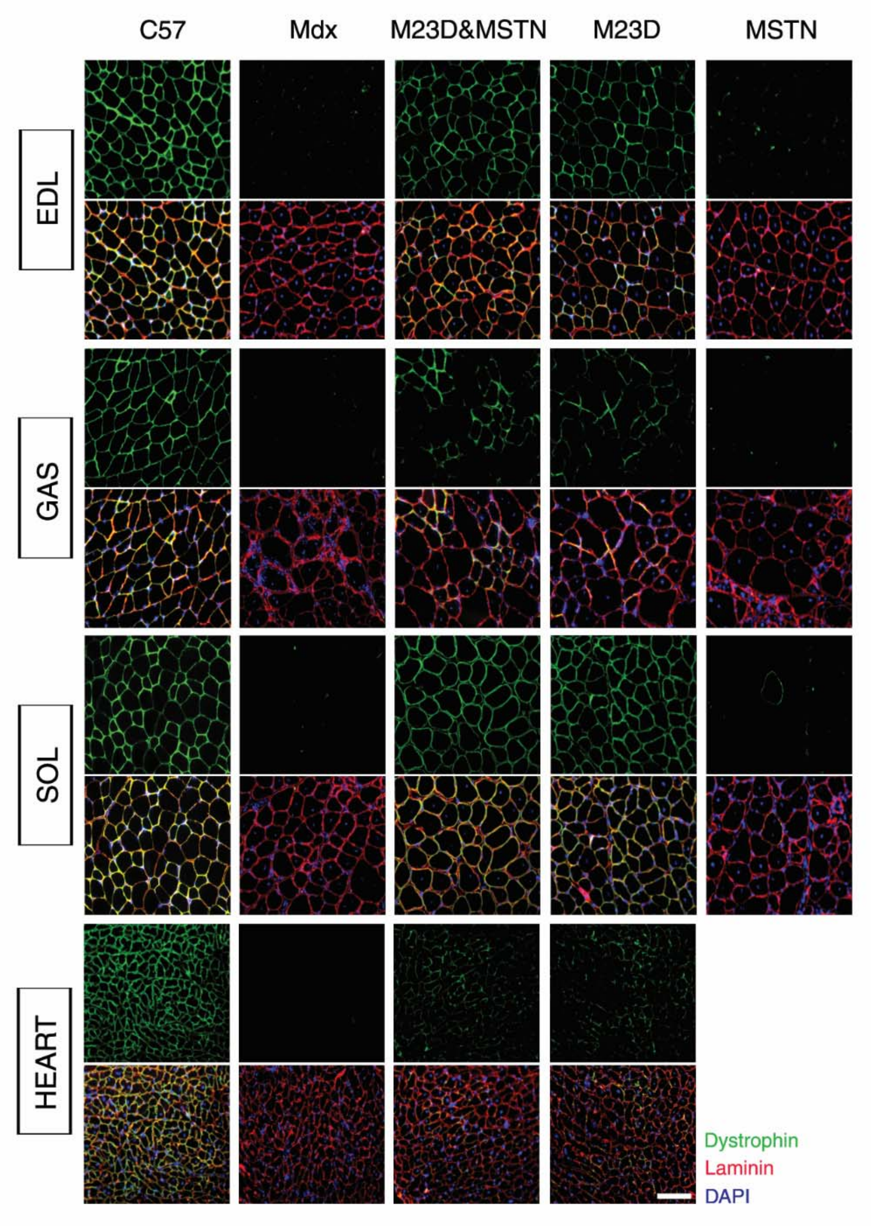

Table S1. Mouse open-field locomotor behaviour

						vs C57				vs Mdx		
**Parameters**	**C57**	**Mdx**	**M23D**	**M23D&MSTN**	**MSTN**	**Mdx**	**M23D**	**M23D& MSTN**	**MSTN**	**M23D**	**M23D& MSTN**	**MSTN**
Total activity	1774 ± 209	1295 ± 136	1279 ± 191	1731 ± 135	1526 ± 172	ns	ns	ns	ns	ns	*↑	ns
Fast activity	360 ± 48	206 ± 29	242 ± 52	282 ± 25	234 ± 30	*↓	ns	ns	*↓	ns	ns	ns
Slow activity	1414 ± 178	1090 ± 113	1038 ± 149	1449 ± 122	1292 ± 155	ns	ns	ns	ns	ns	*↑	ns
Total static counts	1073 ± 133	904 ± 87	821 ± 123	1182 ± 106	1058 ± 134	ns	ns	ns	ns	ns	ns	ns
Fast static counts	67 ± 11	43 ± 5	47 ± 13	64 ± 8	52 ± 9	ns	ns	ns	ns	ns	ns	ns
Slow static count	1006 ± 124	860 ± 82	775 ± 112	1118 ± 99	1007 ± 126	ns	ns	ns	ns	ns	ns	ns
Total mobile counts	701 ± 95	392 ± 54	458 ± 77	549 ± 45	468 ± 57	**↓	ns	ns	*↓	ns	*↑	ns
Fast mobile counts	294 ± 44	163 ± 26	195 ± 42	218 ± 24	183 ± 28	*↓	ns	ns	*↓	ns	ns	ns
Slow mobile counts	408 ± 62	229 ± 33	263 ± 40	331 ± 28	285 ± 35	*↓	ns	ns	ns	ns	*↑	ns
Total rearing counts	1053 ± 319	1022 ± 319	1106 ± 386	1433 ± 276	1060 ± 285	ns	ns	ns	ns	ns	ns	ns
Fast rearing counts	808 ± 335	787 ± 329	886 ± 402	1094 ± 292	751 ± 257	ns	ns	ns	ns	ns	ns	ns
Slow rearing counts	245 ± 28	235 ± 28	221 ± 41	339 ± 33	309 ± 49	ns	ns	*↑	ns	ns	*↑	ns
Total center rearing	67 ± 14	66 ± 6	58 ± 13	121 ± 17	104 ± 23	ns	ns	*↑	ns	ns	**↑	ns
Fast center rearing counts	43 ± 12	43 ± 7	35 ± 9	73 ± 11	69 ± 20	ns	ns	ns	ns	ns	*↑	ns
Slow center rearing	24 ± 6	23 ± 3	23 ± 5	48 ± 10	35 ± 8	ns	ns	ns	ns	ns	*↑	ns
Active time	1079 ± 122	881 ± 81	822 ± 123	1151 ± 90	1025 ± 118	ns	ns	ns	ns	ns	*↑	ns
Static time	758 ± 91	688 ± 61	605 ± 91	888 ± 77	787 ± 98	ns	ns	ns	ns	ns	ns	ns
Mobile time	322 ± 38	193 ± 23	217 ± 36	263 ± 19	237 ± 28	**↓	ns	ns	ns	ns	*↑	ns
Rearing time	1172 ± 205	1117 ± 211	1150 ± 247	1616 ± 165	1242 ± 234	ns	ns	ns	ns	ns	ns	ns
Front to back counts	147 ± 19	98 ± 13	103 ± 16	137 ± 11	120 ± 14	*↓	ns	ns	ns	ns	*↑	ns
Inactive time	2521 ± 122	2720 ± 81	2778 ± 123	2450 ± 90	2576 ± 118	ns	ns	ns	ns	ns	*↓	ns
Distance travelled meters	88 ± 10	67 ± 7	66 ± 12	88 ± 6	77 ± 8	ns	ns	ns	ns	ns	*↑	ns

Using locomotor activity monitors, mouse open-field behavioral activity was assessed during an undisturbed 1 h period per day, for 4 consecutive days. Data were analyzed by Prism5 (GraphPad, San Diego, California, USA) and shown as means ± S.E.M; n = 10 per group. Statistical significance was analyzed by two-tailed Student's t-test (*$p < 0.05$, **$p < 0.01$). Arrows indicate an increase (↑) or decrease (↓) in the value of the parameter of individual groups compared to the control group within each category.
